# Primary adult primitive neuroectodermal tumor of the bladder: A case report and literature review

**DOI:** 10.1097/MD.0000000000021740

**Published:** 2020-08-21

**Authors:** Bing Liu, Dian-Jun Qi, Qing-Fu Zhang

**Affiliations:** a Department of Anorectal Surgery; b Department of General Practice, the First Affiliated Hospital of China Medical University; c Department of Pathology, the First Affiliated Hospital and College of Basic Medical Sciences of China Medical University, Shenyang, PR China.

**Keywords:** adult, case report, neuroectodermal tumor, urinary bladder

## Abstract

**Rationale:**

A primary primitive neuroectodermal tumor (PNET) is a rare and highly malignant tumor that often occurs in the central nervous system of children and young adults. This tumor is rarely observed in the bladder.

**Patient concerns:**

In this paper, we describe the case of a 64-year-old man with a PNET of the bladder. He experienced dull pain in the lower left abdomen for 5 months (without any obvious inducement), which gradually became aggravated and intolerable.

**Diagnoses:**

Partial cystectomy was performed, and a PNET of the bladder, which is extremely rare, was confirmed.

**Interventions:**

Following cystectomy, the patient's general postoperative state was poor and he could not tolerate chemotherapy. Thus, he was subjected to pelvic radiotherapy for 2 weeks.

**Outcomes:**

His physical condition did not improve significantly after radiotherapy; however, we still plan to continue it. If the patient's physical condition improves, chemotherapy will be considered.

**Lessons:**

Most cases of PNETs are intravesical or at least mainly endophytic. However, in this case, the mucosal layer was barely involved, and the tumor mainly grew out of the bladder, which is very rare. The present case provides reference for the diagnosis of PNET.

## 1 Introduction

A primary primitive neuroectodermal tumor (PNET) is a rare and malignant neoplasm that belongs to the Ewing family of tumors. These tumors have a predilection for the central nervous system^[1]^ of children and young adults; however, they may occasionally be observed in other sites such as the chest wall,^[2]^ head and neck,^[3]^ kidneys,^[4]^ adrenal glands,^[5]^ female genital tract,^[6]^ extremities, and scrotal sac.^[7]^ A PNET occurring in the bladder is very rare; it develops more commonly in children and adolescents than in adults. This paper describes a rare case of a PNET in the bladder of an adult.

## 2 Case report

A 64-year-old man (Han Chinese, company staff) presented with complaints of worsening dull pain (that developed 5 months before presentation) in the lower left abdomen. He had not sought any medical intervention before this. Complaints of fever, nausea, and vomiting were denied. He had no personal or family history of tumors. The pain gradually became aggravated and intolerable. While there was tenderness in the lower left abdomen, no rebound pain or muscle tension was observed.

**Figure 1:**
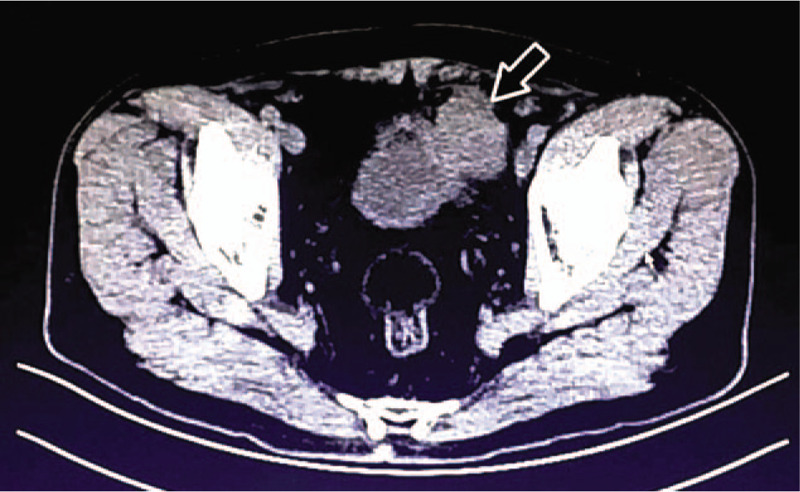
Pelvic computed tomography scan. The tumor is located on the left lateral wall of the bladder (arrow). It has the same density as the bladder wall, an unclear margin, and a size of about 6 × 5 cm. Its boundary with the surrounding tissues is unclear.

No obvious abnormalities were found in the laboratory examinations. Abdominal computed tomography (CT) upon admission revealed that a pelvic tumor had developed in the left lateral wall of the bladder and measured about 6 × 5 cm (Fig. 1). No significant abnormality was found by cystoscopy. No tumor metastasis was detected by head CT, bone scan, abdominal ultrasonography, and lung radiography.

**Figure 2:**
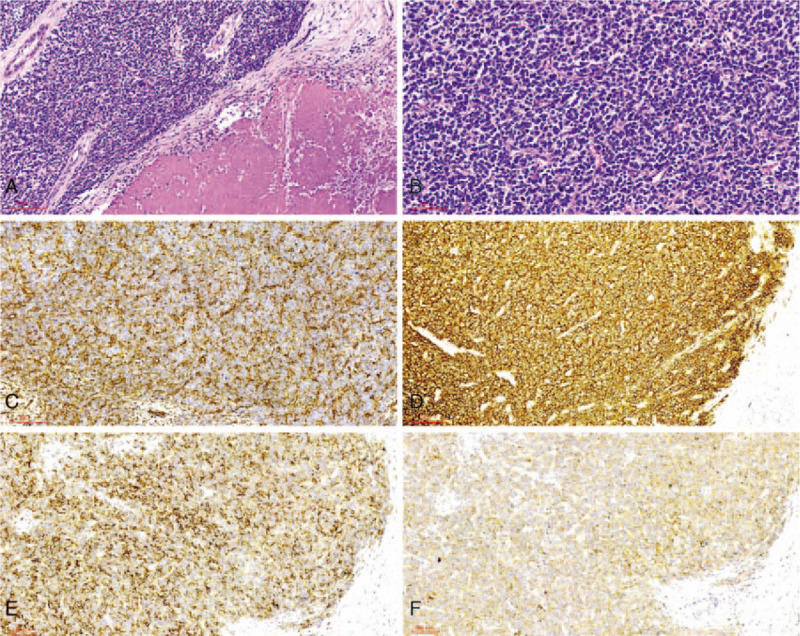
HE staining and immunohistochemical staining. A: Small, round tumor cells are distributed in a patchy form; they have a similar cell size and morphology, little cytoplasm that stains light, deeply stained nuclei, easily visible mitotic figures, and large areas of local necrosis (HE staining; magnification: ×100). B: At high magnification, the tumor cells are seen to form Homer-Wright rosettes (HE staining; magnification: ×200). C: Immunohistochemistry shows that it is a vimentin-positive diffuse-type lesion (magnification: ×100). D: Immunohistochemistry shows a diffuse-type lesion with strong CD99 positivity (magnification: ×100). E: Immunohistochemistry shows a CD56-positive diffuse-type lesion (magnification: ×100). F: Immunohistochemistry shows a synaptophysin-positive diffuse-type lesion (magnification: ×100). HE = Hematoxylin and eosin.

A histopathological examination revealed that the tumor consisted of densely distributed, small-to-medium sized, similar round cells. The nucleus-cytoplasm ratio was high, and the chromatin had a fine texture. Nuclear division was observed frequently and was accompanied by massive coagulative necrosis. Homer-Wright rosettes (Fig. 2A, B) were observed occasionally. Immunohistochemical examination revealed that the tumor cells expressed vimentin, CD99, synaptophysin, and CD56. The cells stained negative for CK (a broad-spectrum marker), P63, GATA3, NKX3.1, PSA, and S-100, and the Ki-67 proliferation index was nearly 50% (Fig. 2C–F). Fluorescence in situ hybridization detected the following gene mutations: *t*(11;22) (q24;Q12) chromosome translocation and *EWS-FL1* fusion gene expression (type 1 subtype). Thus, based on the results of the immunohistochemical examination and fluorescence in situ hybridization, a PNET of the bladder was diagnosed.

The patient underwent surgery. Intraoperative exploration revealed that the tumor had grown on the left wall of the bladder and was located close to the left pelvic wall. It had a hard, crispy texture and an unclear boundary. It was greyish white and red patches in color and had not become saturated with the bladder mucosa. The posterior wall of the bladder was separated from the peritoneum, and partial cystectomy was performed. The patient's general postoperative state was poor, and he could not tolerate chemotherapy. Presently, he has received 2 weeks of pelvic radiotherapy. His physical condition has not improved significantly and he is still unable to tolerate chemotherapy. We plan to continue radiotherapy; if his physical condition improves, chemotherapy will be considered.

The study was reviewed and approved by the Ethics Committee of the First Affiliated Hospital of China Medical University.

## 3 Discussion

PNETs originate from neural crest cells^[7]^ and can be classified into central PNETs and peripheral PNETs (pPNETs). Most patients with PNETs have a young age at onset. However, according to the 9 cases of bladder PNETs (Table 1) currently available in the literature,^[8–16]^ this type of PNET is predominant in middle-aged and elderly individuals, with an average onset age of 53.5 years; in 60% of the cases, including the present one (6/10), the patients were older than 60 years. This type of bladder tumor is often overlooked by clinicians, because the patient's age and tumor site show inconsistencies with the onset characteristics of PNETs.

**Table 1:**
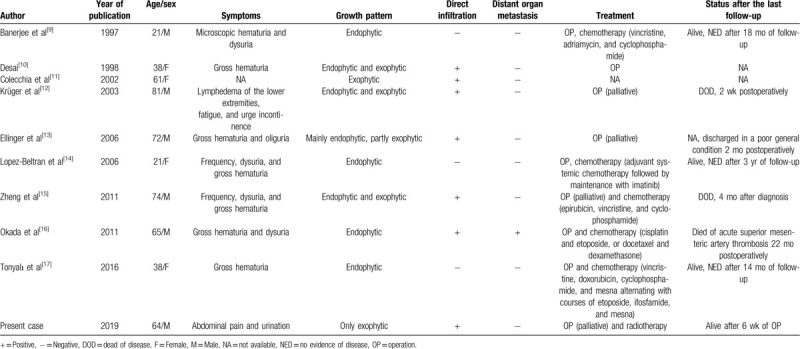
Characteristics of 10 cases of primitive neuroectodermal tumors of the bladder.

Ninety percent (9/10) of the cases of adult bladder PNETs reported endophytic growth; 4 of these were accompanied by an exophytic growth. Under such conditions, the lesion can be located preoperatively by cystoscopy, and pathological examination can help make a definite diagnosis. The tumor in the present case showed a purely exophytic growth. No significant abnormality was found by cystoscopy. Intraoperatively, the bladder mucosal layer was found to be smooth, and no tumor definitely involving the mucosal layer was found. Therefore, it was not possible to locate the lesion or make a definite diagnosis on the basis of cystoscopic findings. pPNETs originate from the neural crest cells outside the central nervous system.^[17]^ In this case, the PNET grew in the bladder and barely involved the mucosal layer, because of the presence of neural crest cells in the bladder submucosa or muscular layer. Therefore, preoperative ultrasonography or CT was necessary. These modalities can avoid a missed diagnosis and can help in determining the anatomical relationship between the tumor and its surrounding tissues and whether there were enlarged lymph nodes around it, thus providing a basis for the formulation of treatment plans. In recent years, percutaneous fine-needle puncture guided by ultrasonography or CT has been applied frequently, which is helpful for making an early diagnosis of a PNET.

Generally, the prognosis of PNETs is poor^[15]^; the prognosis for adults is worse than that for minors.^[18]^ The survival rates of patients with a pPNET or central PNET are roughly equivalent.^[19]^ The prognosis of PNETs in the head and neck is better than that in other parts of the body. Tumors with a soluble *EWS-FLI1* gene have a better prognosis than those with less soluble genes. In 8 cases of adult bladder PNET reported before, the frequency of localized infiltration and growth was more (7/8) than that of remote metastasis (1/8). Patients with local infiltration can rarely undergo a radical operation (RO); hence, they can only undergo palliative surgery, which is associated with a poor prognosis. Therefore, the presence of local infiltration is a sign of poor prognosis in cases of adult bladder PNET. The only reported case of multiple metastases occurred 8 months postoperatively. The patient died after 22 months from acute superior mesenteric artery thrombosis. Because of the small sample size and a short follow-up in some cases, it is not possible to make a comprehensive and objective evaluation of the prognostic factors of PNETs of the bladder.

Currently, surgery is the main treatment for PNETs, and it should be performed in patients who can tolerate it.^[8,9,11–16]^ The prognosis of patients whose tumors can be completely removed (complete RO) is better than that of those who undergo palliative surgery.^[8–16]^ Postoperative chemotherapy is the conventional treatment for adult bladder PNETs; vincristine, adriamycin, cyclophosphamide, epirubicin, cisplatin, etoposide, docetaxel, dexamethasone, doxorubicin, and mesna have been used as chemotherapeutic agents in such cases.^[8,12–16]^ The symptoms in most patients were relieved to varying degrees after chemotherapy. Radiotherapy has been also used for local disease control in pPNETs,^[20,21]^ and it is applicable to patients who have not undergone complete RO. The patient herein had a poor postoperative condition and could not tolerate the side effects of chemotherapy. Therefore, pelvic radiotherapy was chosen. After radiotherapy, the patient's abdominal pain, mental status, and strength improved slightly, but he could still only barely tolerate chemotherapy. Because of the short follow-up, the efficacy of radiotherapy for bladder PNETs needs further investigation.

## 4 Conclusion

Usually, PNETs are intravesical or at least mainly endophytic. However, the mucosal layer was barely involved occasionally, and the tumor mainly grew out of the bladder. This case provides reference for the diagnosis of PNET in the future.

## Acknowledgments

We would like to thank Editage (www.editage.cn) for English language editing.

## Author contributions

**Data curation:** Bing Liu, Dian-Jun Qi, Qing-Fu Zhang.

**Formal analysis:** Bing Liu.

**Methodology:** Bing Liu, Dian-Jun Qi, Qing-Fu Zhang.

**Resources:** Bing Liu, Dian-Jun Qi, Qing-Fu Zhang.

**Software:** Bing Liu.

**Supervision:** Dian-Jun Qi.

**Writing – original draft:** Bing Liu.

**Writing – review & editing:** Dian-Jun Qi, Qing-Fu Zhang.

## References

[1] Gao J Chow E Aloma A Peripheral primitive neuroendocrine tumor of the chest wall – a case report with pathological correlation. Radiol Case Rep 2018;13:392–6. 29904480 10.1016/j.radcr.2018.01.003 PMC6000041

[2] Rahbar M Rahbar M Bahoush G Peripheral primitive neuroectodermal tumor associated with paraneoplastic Cushing's syndrome: the rare case. Ann Med Surg (Lond) 2018;37:21–4. 30581565 10.1016/j.amsu.2018.11.018 PMC6287080

[3] Parcesepe P Giordano G Zanella C Colonic Ewing Sarcoma/PNET associated with liver metastases: a systematic review and case report. Pathol Res Pract 2019;215:387–91. 30553605 10.1016/j.prp.2018.11.021

[4] Celli R Cai G Ewing sarcoma/primitive neuroectodermal tumor of the kidney: a rare and lethal entity. Arch Pathol Lab Med 2016;140:281–5. 26927724 10.5858/arpa.2014-0367-RS

[5] Dai J He HC Huang X Long-term survival of a patient with a large adrenal primitive neuroectodermal tumor: a case report. World J Clin Cases 2019;7:340–6. 30746375 10.12998/wjcc.v7.i3.340 PMC6369397

[6] Chiang S Snuderl M Kojiro-Sanada S Primitive neuroectodermal tumors of the female genital tract: a morphologic, immunohistochemical, and Molecularn study of 19 cases. Am J Surg Pathol 2017;41:761–72. 28296680 10.1097/PAS.0000000000000831 PMC5525138

[7] Baleato-González S Tirapu-de-Sagrario MG Pintos-Martínez E Scrotal peripheral primitive neuroectodermal tumor. Curr Urol 2018;12:50–3. 30374281 10.1159/000447231 PMC6198772

[8] Banerjee SS Eyden BP McVey RJ Primary peripheral primitive neuroectodermal tumour of urinary bladder. Histopathology 1997;30:486–90. 9181373 10.1046/j.1365-2559.1997.00524.x

[9] Desai S Primary primitive neuroectodermal tumour of the urinary bladder. Histopathology 1998;32:477–8. 9639125 10.1046/j.1365-2559.1998.0358a.x

[10] Colecchia M Dagrada GP Poliani PL Immunophenotypic and genotypic analysis of a case of primary peripheral primitive neuroectodermal tumour (pPNET) of the urinary bladder. Histopathology 2002;40:108–9. 11903609 10.1046/j.1365-2559.2002.1340e.x

[11] Krüger S Schmidt H Kausch I Primitive neuroectodermal tumor (PNET) of the urinary bladder. Pathol Res Pract 2003;199:751–4. 14708642 10.1078/0344-0338-00492

[12] Ellinger J Bastian PJ Hauser S Primitive neuroectodermal tumor: rare, highly aggressive differential diagnosis in urologic malignancies. Urology 2006;68:257–62. 16904430 10.1016/j.urology.2006.02.037

[13] Lopez-Beltran A Pérez-Seoane C Montironi R Primary primitive neuroectodermal tumour of the urinary bladder: a clinico-pathological study emphasising immunohistochemical, ultrastructural and molecular analyses. J Clin Pathol 2006;59:775–8. 16803953 10.1136/jcp.2005.029199 PMC1860413

[14] Zheng Y Tan F Wang L Primary primitive neuroectodermal tumor of the urinary bladder: a case report and literature review. Med Oncol 2011;Suppl 1: S388–391. 20852971 10.1007/s12032-010-9680-3

[15] Okada Y Kamata S Akashi T Primitive neuroectodermal tumor/Ewing's sarcoma of the urinary bladder: a case report and its molecular diagnosis. Int J Clin Oncol 2011;16:435–8. 21063743 10.1007/s10147-010-0144-8

[16] Tonyali Ş Yazici S Yeşilirmak A The Ewing's sarcoma family of tumors of urinary bladder: a case report and review of the literature. Balkan Med J 2016;33:462–6. 27606145 10.5152/balkanmedj.2016.16533 PMC5001827

[17] Kumar V Singh A Sharma V Primary intracranial dural-based Ewing sarcoma/peripheral primitive neuroectodermal tumor mimicking a meningioma: A rare tumor with review of literature. Asian J Neurosurg 2017;12:351–7. 28761507 10.4103/1793-5482.185060 PMC5532914

[18] de Alava E Kawai A Healey JH EWS-FLI1 fusion transcript structure is an independent determinant of prognosis in Ewing's sarcoma. J Clin Oncol 1998;16:1248–55. 9552022 10.1200/JCO.1998.16.4.1248

[19] Kampman WA Kros JM De Jong TH Primitive neuroectodermal tumours (PNETs) located in the spinal canal; the relevance of classification as central or peripheral PNET: case report of a primary spinal PNET occurrence with a critical literature review. J Neurooncol 2006;77:65–72. 16292490 10.1007/s11060-005-9006-z

[20] Wu XL Dai YJ Sun GY Adult neuroblastoma in the retroperitoneum: a case report. Medicine (Baltimore) 2018;97:e13750. 30572519 10.1097/MD.0000000000013750 PMC6320202

[21] Sueyoshi R Okawada M Fujimura J Successful complete resection of Ewing sarcoma arising from the bladder in a 10-year-old boy after chemotherapy. Pediatr Surg Int 2014;30:965–9. 25080033 10.1007/s00383-014-3573-z

